# Assessment of Salivary MMP-8 and IL-1β for the Diagnosis of Periodontal Diseases in Pakistani Population

**DOI:** 10.1055/s-0043-1772779

**Published:** 2023-12-12

**Authors:** Rida Kainat, Iftikhar Ahmed, Abdullah Mohammed Alolaywi, Humera Waheed, Zohaib Khurshid Sultan, Syed Faraz Moin

**Affiliations:** 1 Department of Biochemistry, Baqai Medical University, Karachi, Pakistan; 2 Dental Department, Hotat Bani Tamim General Hospital, Ministry of Health, Riyad, Saudi Arabia; 3 Dow College of Biotechnology, Dow University of Health Sciences, Karachi, Pakistan; 4 Department of Prosthodontics and Dental Implantology, College of Dentistry, King Faisal University, Al-Ahsa, Saudia Arabia; 5 Department of Anatomy, Faculty of Dentistry, Center of Excellence for Regenerative Dentistry, Chulalongkorn University, Bangkok, Thailand; 6 Dr. Zafar H. Zaidi Center for Proteomics, University of Karachi, Karachi, Pakistan

**Keywords:** saliva, biomarkers, MMP-8, IL-1β, gingivitis, periodontal disease

## Abstract

**Objective** Clinical methods use the subjective diagnosis of periodontal diseases by visual observation that could result in differences and variability of diagnosis. The addition of specific markers could aid in the accurate diagnosis of the local population. The objective of the study was to target two of the major proteins for possible significance in such an approach.

**Materials and Methods** Unstimulated saliva samples were collected from 60 participants aged between 18 and 70 years. Three groups each with twenty participants were recruited into periodontitis, gingivitis, and healthy control.

**Statistical Analysis** The samples were analyzed using human enzyme-linked immunosorbent assay kits for matrix metalloproteinase-8 (MMP-8) and interleukin-1β (IL-1β).

**Results** SPSS version 20 was used to analyze the result. Posthoc analysis by Tukey's test revealed that MMP-8 levels were higher in gingivitis and periodontitis groups as compared with healthy controls. The test also revealed that IL-1β levels were higher in the periodontitis group compared with the healthy control and gingivitis group. Additionally, one-way analysis of variance analysis showed a significant effect on probing depth in gingivitis and periodontitis patients. The mean age of periodontitis group was significantly higher than other groups.

**Conclusion** Salivary biomarkers may provide useful diagnostic information and could be utilized as tests for periodontal disease screening, prognosis, and prediction.

## Introduction

Periodontal problems brought on by plaque are referred to as gingivitis and periodontitis, respectively. The term “periodontitis” refers to infections that affect the periodontium. If untreated, periodontal illnesses can develop into an irreversible condition that damages the complex periodontal tissues. 1 A full mouth examination, which includes probing pocket depth and gingival attachment levels, is used to diagnose periodontal disease. 2 Subjective clinical methods are used to identify gingivitis and periodontitis. Since both disorders are identified with a dental probe, the World Health Organization probe (WHO) also known as The Community Periodontal Index of Treatment Needs (CPITN) probe is routinely used. The diagnosis varies depending on the examiner. This results from variations in the probe's design, angulation, and examiner pressure. Achieving satisfactory results also depends on the patient's tolerance. As a result, the outcome may differ, which is referred to as measurement inaccuracy owing to subjectivity. Although the manual periodontal probe is the most cost-effective and straightforward diagnostic tool, it is futile when accurate diagnosis and uniformity are required. 3 Bleeding on probing, which is used to suggest local inflammation, is not a useful indicator of disease activity or tissue damage. 4 For example, only approximately 30% of regions that bleed when probed on subsequent inspections are likely to progress. 5 In recent decades, salivary component diagnosis has received a lot of consideration. Salivaomics is an interdisciplinary study of saliva's contents, functions, and methodologies. 6 7 8 Saliva is not a uniform fluid since it contains a range of biomolecules. These include nucleic acids such as DNA and RNA, in conjunction with organic molecules, especially the proteins. It is a part of the neuroendocrine system that holds potential biomarkers for diseases. These biomarkers can help with the diagnosis and treatment of certain acute and chronic inflammatory diseases as well. The saliva offers some positive distinct features such as ease of collection, painlessness, non-invasive, high patient compliance, effortlessness, and nontoxic if held with care. 9

Ventures like human salivary proteome project provide a better understanding toward disease pathogenesis and examine the impact of medications on salivary proteins. 10 Periodontal degradation is mediated from a variety of mediators especially cytokines and other protein such as periostin. These are messenger molecules made up of soluble proteins that interact with other cells and tend to set off a cascade of proceeding pathways that increase the inflammatory reaction, stimulate the creation of enzymes that break down connective tissue, and drive osteoclastic bone resorption. 11 12 Although both types of interleukin-1 (IL-1; IL-1α and IL-1β have similar biological functions, IL-1β is more effective at promoting osteoclast activity and is more commonly detected in periodontitis. 13 Some collagenases such as matrix metalloproteinases (MMPs; MMP-1, -8, -13, and -14) and gelatinase MMPs (MMP-2 and -9) play a significant role in periodontal degeneration due to their collagen-degrading properties. Natural collagen I and III are destroyed by gelatinases, which are then degraded by collagenases. MMP-8 is among the most prominent MMPs involved in the deterioration of periodontal tissue. 14

Salivary indicators such IL-1β, IL-6, MIP-1, and MMP-8 in saliva of 209 patients were detected in the year 2015. The study found that these biomarkers could tell the difference between healthy gingiva and periodontal disease. 15 Biomarkers were looked at and discovered in a research with the goal of enhancing periodontitis diagnostic standards to go beyond periodontal probe-based diagnosis. The study examined and compared the effectivity and accuracy rate of eight biomarkers in diagnosing gingivitis using receiver operating characteristic curves. 16

Since it has been deduced over time that biomarker discovery cannot only help in identification of periodontal diseases in the saliva but also aid in diagnostics related to gingival crevicular fluid (GCF) using proteomic analysis. 17 Moreover, these inflammatory markers are mostly generated in GCF, although they ultimately flow out and become part of the saliva. 18 To validate the clinical diagnosis of periodontitis and gingivitis in the local population, it is necessary to study a combined periodontal status model that accounts for both periodontitis and gingivitis and to examine these particular salivary biomarkers.

## Materials and Methods

The study population comprised of 60 medically healthy participants aged between 18 and 70 years participated in the study conducted by the Department of Biochemistry, Baqai Dental College, Baqai Medical University located in Karachi, Pakistan. Formal consent was taken from all the participants.

Twenty participants each were recruited into periodontitis, gingivitis, and healthy control groups established on oral examination results. Patient periodontal status was classified according to the CPITN. This study was conducted with approval from Ethics and Board of Advanced Studies and Research (BASR, IBC # BMU-EC/02–2021) and the samples were analyzed at the National Institute of Proteomics, Karachi University.

### Inclusion Criteria

1. Healthy individuals (both males and females) aged 18 and above, without any prior periodontal, systemic, or bone disorders.
2. Diagnosed patients with clinical and radiographic findings of gingivitis and periodontitis.

### Exclusion Criteria 19

1. Individuals with any systemic illness or medical conditions that can have a direct influence on the periodontium or salivary pH such as diabetes mellitus, hypertension, renal or hepatic diseases, arthritis, salivary gland diseases, malignancies or immunodeficiency, denture wearers, current/past tobacco users, patients on antibiotics or those who had taken in past three months, antifungal, or immunosuppressive medications are excluded from the study.
2. Pregnant and breastfeeding females.
3. Patients on antibiotics, vitamin D, or calcium supplements for the last 3 months.
4. Medically compromised patients with genetic defects, physiological, or bone disorders.
5. Patients who had undertaken any periodontal treatment within the last 6 months.

### Clinical Parameters

Individuals were evaluated in an outpatient department in natural daylight while sitting on a dental chair. The CPITN index for measuring the pocket depth was used. 20 On gently probing, a sterile CPITN probe was utilized to observe bleeding. Cotton wool rolls were used to dry the teeth when necessary.

Between examinations, the dental chairs were disinfected. A visual examination was performed first to assess the individual's oral health. Wet teeth were examined, and food material if present was removed with a CPITN probe. Following that, the soft tissues and oral cavity were inspected for any abnormalities. Precautions were taken at all times. All examiners and recorders in attendance had personal protective clothing and equipment. Each individual was examined with latex-free examination gloves, which were replaced before moving on to the next patient. A facemask was worn at all times and was replaced frequently. Each set of tools was placed on a disposable paper sheet, which was discarded after each inspection.

The CPITN probes and mirrors were transported in a container designed specifically for “contaminated” instruments. At the end of each session, all reusable instruments were cleansed and autoclaved. In accordance with infection control best practice, all contaminated waste, including gloves, facemasks, cotton wool rolls, tissues, and wipes, was disposed of in yellow “hazardous waste” bags. Two plastic instrument boxes were used, one for sterile equipment exclusively and the other for contaminated instruments.

### Collection of Unstimulated Saliva

Unstimulated saliva was collected by drooling into a falcon conical tube (15 mL) using a funnel with the participant's head tilted forward slightly in a sitting position on a dental chair. 21 The sampling lasted 15 minutes and ended when the total amount collected reached 5 mL. Each collected sample was then placed on ice immediately. Afterward the samples were cold centrifuged in a centrifuge (ThermoFisher, Heraeus Megafuge 8R, Zweigniederlassung Osterode, Am Kalkberg, 37520. Osterode am Harz. Germany) for 15 minutes at high speed (7,000 rpm) to remove any solid particles from the oral cavity. The supernatant was separated and stored at low temperature (-20°C) until further analysis. 22 This study was approved by the Institutional Ethical Committee, Baqai Medical University Karachi (BASR, IBC # BMU-EC/02–2021). Informed consent was obtained from each participant before proceeding with the study.

### Protein Biomarker Assay

Biomarker levels were detected with enzyme-linked immunosorbent assays for measurement of active MMP-8 (human MMP-8 activity assay) and IL-1β (human interleukin-1 beta assay) according to the manufacturers' protocols. Both the kits used the sandwich method. Tests enzyme-linked immunosorbent assay kit for MMP-8 (USCN, Cat. No. SEA103Hu, Wuhan USCN Business Co., Ltd, China) and (USCN, Cat. No. SEA563Hu, Wuhan USCN Business Co., Ltd, China) enzyme-linked immunosorbent assay kit for IL-1β with the expiry date of February 2022, Lot numbers: L210625801 and L20602367, respectively, were used according to the given methodology.

### Data Analysis

All information was analyzed by computer database by using IBM SPSS Statistics 20.

## Results

**Fig. 1:**
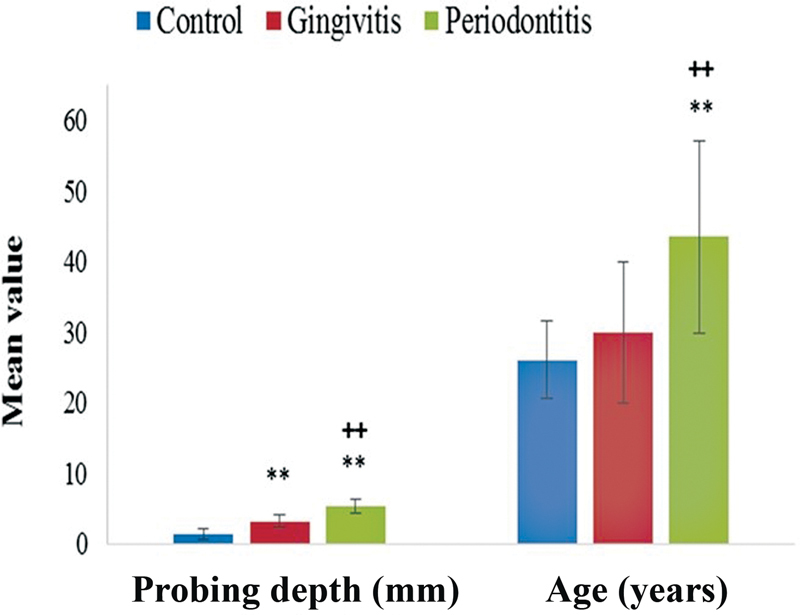
Comparison of probing depth (mm) and age (years) among healthy, gingivitis, and periodontitis groups. Data represented as mean value ± standard deviation ( *n* = 20). Significantly different by Tukey's test: ** *p* < 0.01 from control group; ++ *p* < 0.01 from gingivitis group.

Sixty persons were enrolled in this study, out of which 20 (33.33%) were healthy individuals (controls), 20 (33.33%) gingivitis subjects, and 20 (33.33%) periodontitis subjects. Table 1 presented the comparison of baseline parameters among healthy, gingivitis, and periodontitis subjects. Data analysis by one-way analysis of variance (ANOVA) showed significant effect on probing depth (F = 58.279, df = 2,57, *p* < 0.01). The probing depth was higher in gingivitis and periodontitis group as compared with healthy control group (1.4 ± 0.69 vs. 3.23 ± 0.86 mm; *p* -value <0.01 and 1.4 ± 0.69 vs. 5.32 ± 1.1 mm; *p* -value <0.01), respectively. One-way ANOVA showed significant effect of age (F = 9.691, df = 2,57, *p* < 0.01). The mean age of periodontitis group was significantly higher than healthy subjects (26 ± 5.47 vs. 43.5 ± 14.16 years; *p* < 0.01) and gingivitis group (30 ± 9.96 vs. 43.5 ± 14.16 years; *p* < 0.01; Fig. 1 ).

**Table 1:** Comparison among healthy, gingivitis, and periodontitis subjects for the correlation of age and the mean probing depth

Variables	Control	Gingivitis	Periodontitis	*p* -Value
Number of individuals	20 (33.33%)	20 (33.33%)	20 (33.33%)	–
Mean age (years)	26 ± 5.47	30 ± 9.96	43.5 ± 14.16	*p* < 0.01
Mean probing depth (mm)	1.4 ± 0.69	3.23 ± 0.86	5.32 ± 1.1	*p* < 0.01

The demographic data showed that periodontitis was prevailing in males as compared with female where gingivitis was predominant. The mean age conversely falls in above 40 years for periodontitis for both the gender. For gingivitis, the mean age for males was 30 years, while that for females it was 26 years. Table 2 presents the details of data.

**Table 2:** The gender wise demographic details of individuals included in this study

Variables		Total ( *n* = 60)	Control	Gingivitis	Periodontitis
Gender	Male	32 (53.33%)	6 (30%)	12 (60%)	14 (70%)
	Female	28 (46.66%)	14 (70%)	8 (40%)	6 (30%)
Age (years)	Male	36.4 ± 14	17.5 ± 1	30 ± 10.9	41 ± 14.8
	Female	30.5 ± 10.8	27 ± 6.2	26.5 ± 7.8	42.25 ± 14

Sixty persons were enrolled in this study, out of which 32 (53.33%) were male and 28 (46.66%) were female subjects. In control group, 6 (30%) were male and 14 (60%) were females. Gingivitis group has 12 (60%) male and 8 (40%) females. In periodontitis group, 14 (70%) were males and 6 (30%) females.

### MMP-8 Levels

**Fig. 2:**
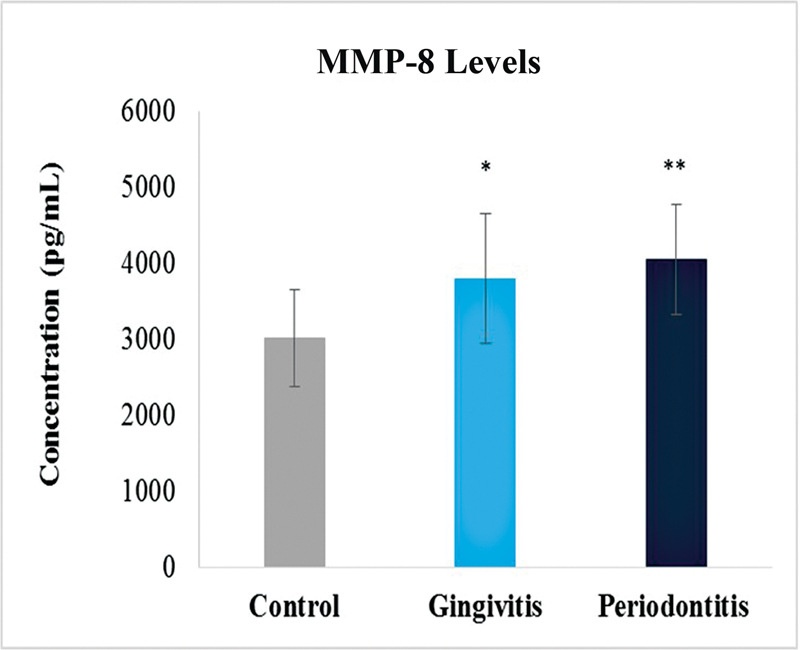
Levels of matrix metalloproteinase-8 (MMP-8) in control, gingivitis, and periodontitis groups. Significantly different by Tukey's test: * *p* < 0.01 from control group; ** *p* < 0.01 from gingivitis group.

**Fig. 3:**
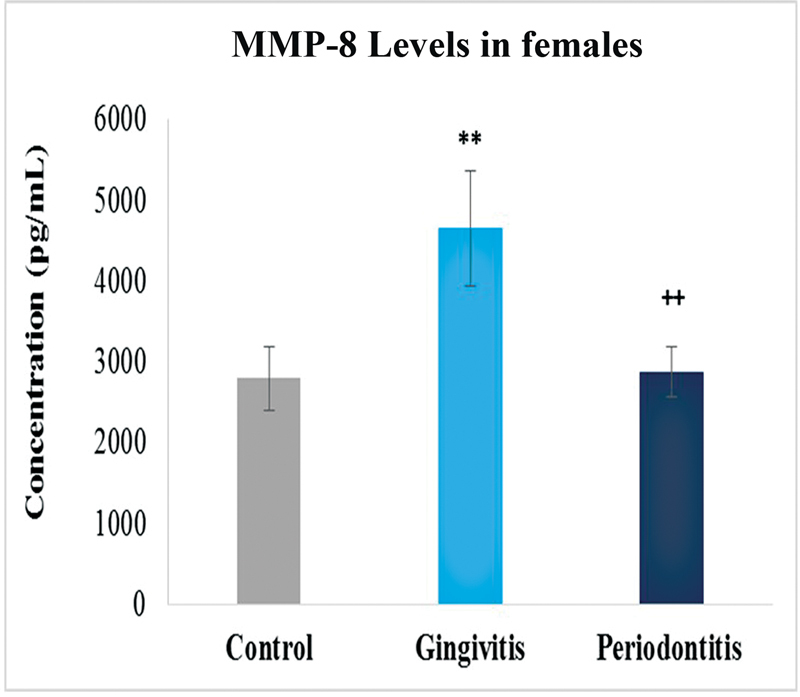
Levels of matrix metalloproteinase-8 (MMP-8) in control, gingivitis and periodontitis groups of females. Significantly different by Tukey's test: ** *p* < 0.01 from control group; ++ *p* < 0.01 from gingivitis group.

**Fig. 4:**
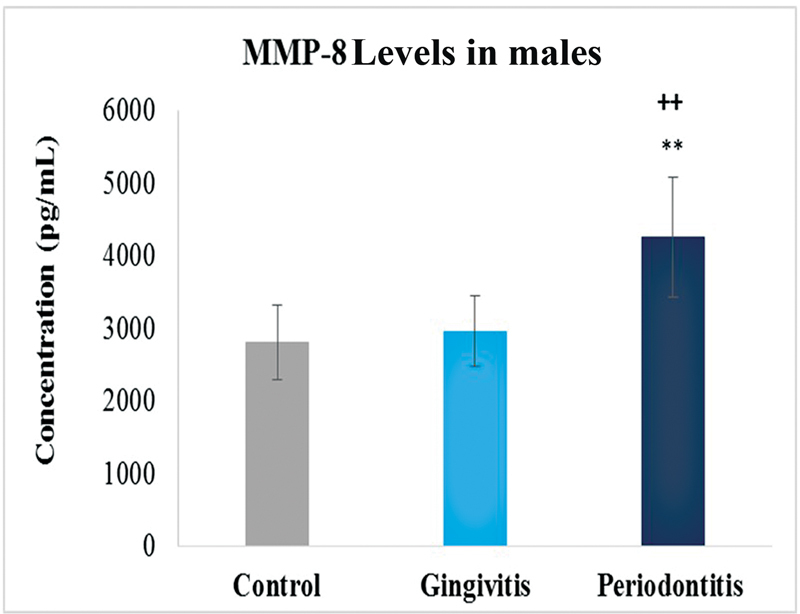
Levels of matrix metalloproteinase-8 (MMP-8) in control, gingivitis and periodontitis groups of males. Significantly different by Tukey's test: ** *p* < 0.01 from control group; ++ *p* < 0.01 from gingivitis group.

The MMP-8 levels were compared between the three groups. There was a significant difference between the values of the MMP-8 levels of control group and disease groups. Highest levels were observed in periodontitis group followed by gingivitis group. The concentrations were measured in picograms per milliliter ( Fig. 2 ). Interesting results were observed when the values were compared with respect to gender. Female samples showed high levels of MMP-8 in gingivitis. On the other hand, male samples showed high levels in periodontitis as compared with the control group that was constant in all comparisons in MMP-8 analysis ( Figs. 3 and 4 ).

### IL-1β Levels

**Fig. 5:**
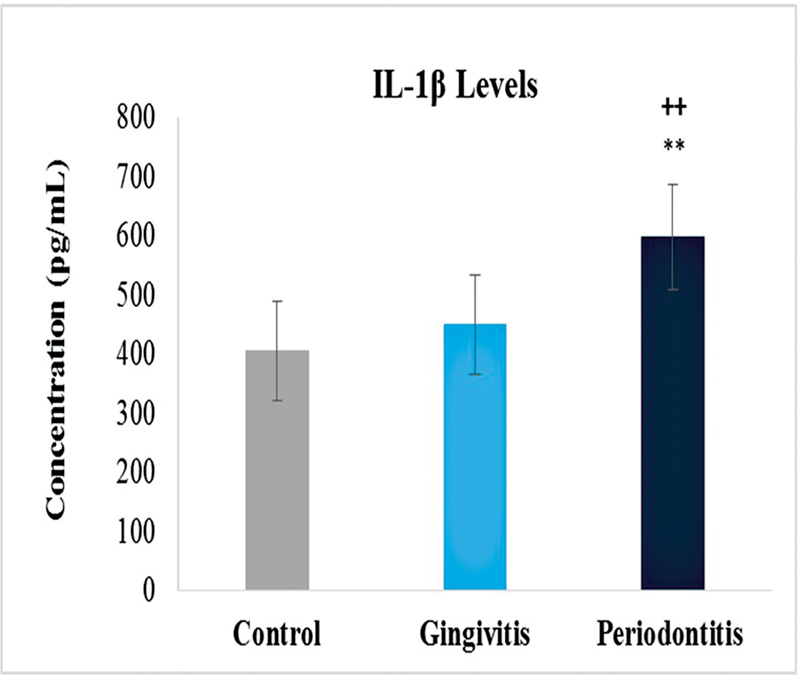
Levels of interleukin-1β (IL-1β) in control, gingivitis and periodontitis groups. Significantly different by Tukey's test: *p* < 0.01 from control group; ** *p* < 0.01 from periodontitis group.

**Fig. 6:**
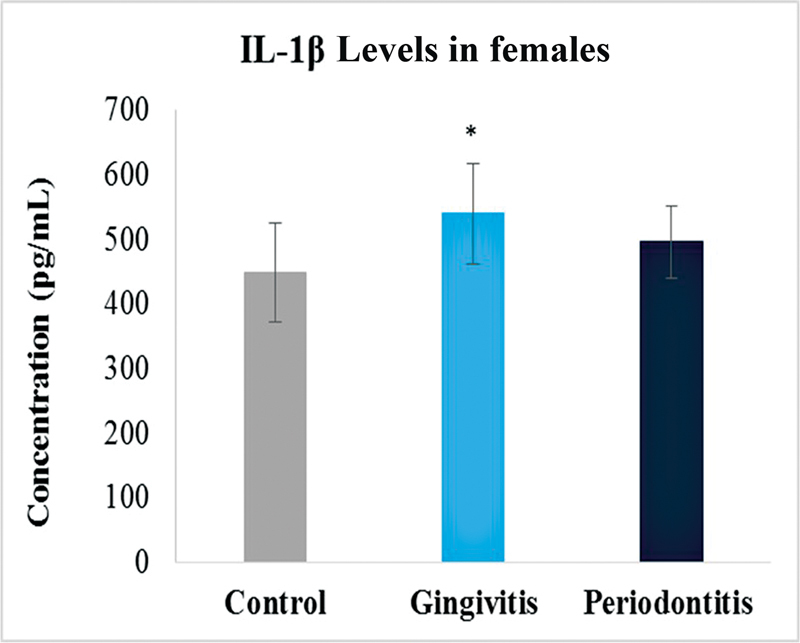
Levels of interleukin-1β (IL-1β) in control, gingivitis and periodontitis groups in females. Significantly different by Tukey's test: *p* < 0.01 from control group; * *p* < 0.01 from gingivitis group.

**Fig. 7:**
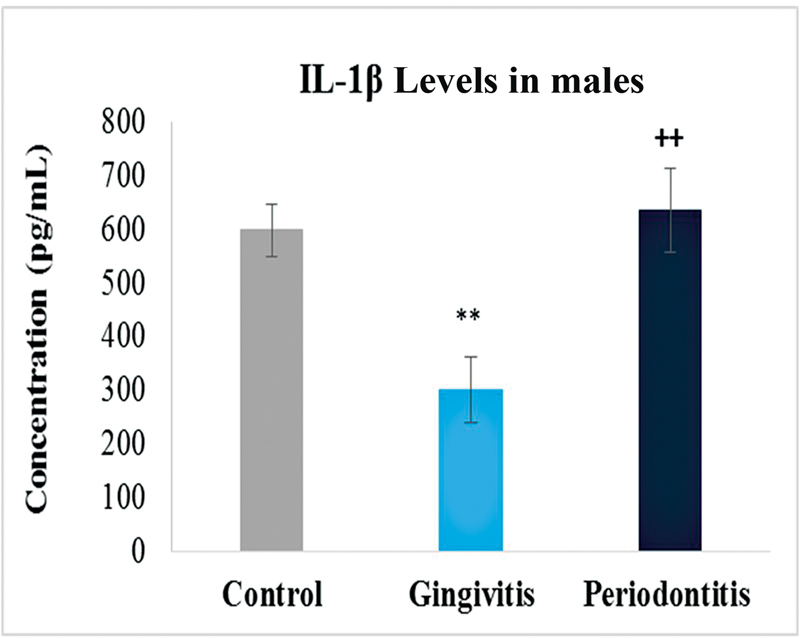
Levels of interleukin-1β (IL-1β) in control, gingivitis and periodontitis groups in males. Significantly different by Tukey's test: *p* < 0.01 from control group; ** *p* < 0.01 from gingivitis group.

Similarly, the levels of IL-1β were also compared between the three groups. There was a significant difference observed between the values of the control group and disease groups. Highest levels were observed in periodontitis group just as with the MMP-8 followed by gingivitis group ( Fig. 5 ). Gingivitis group showed increased levels as compared with control but was not that significant. With respect to gender, female gingivitis samples showed high levels of IL-1β with respective control. On the other hand, male samples showed slightly high levels in periodontitis, while low levels in gingivitis samples with respective control ( Figs. 6 and 7 ).

### Bivariate Pearson Correlation between Age, PD, MMP-8, and IL-1β

**Fig. 8:**
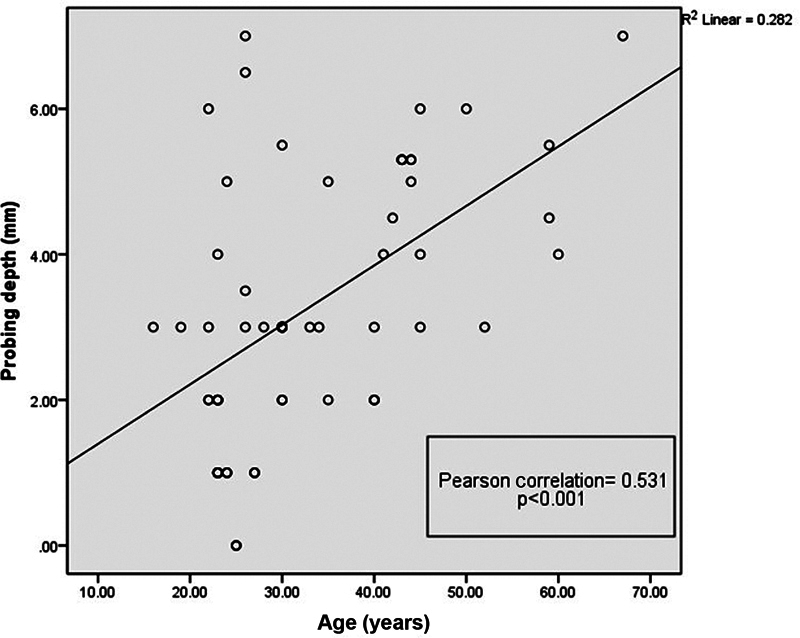
Age had a positive correlation with probing depth ( *p* -value < 0.001).

The correlation analysis showed that there is a positive correlation of age and the depth of periodontal pocket ( Fig. 8 ). The analysis in Table 1 presented the values that showed significant increase in probing depth of periodontitis patients.

**Fig. 9:**
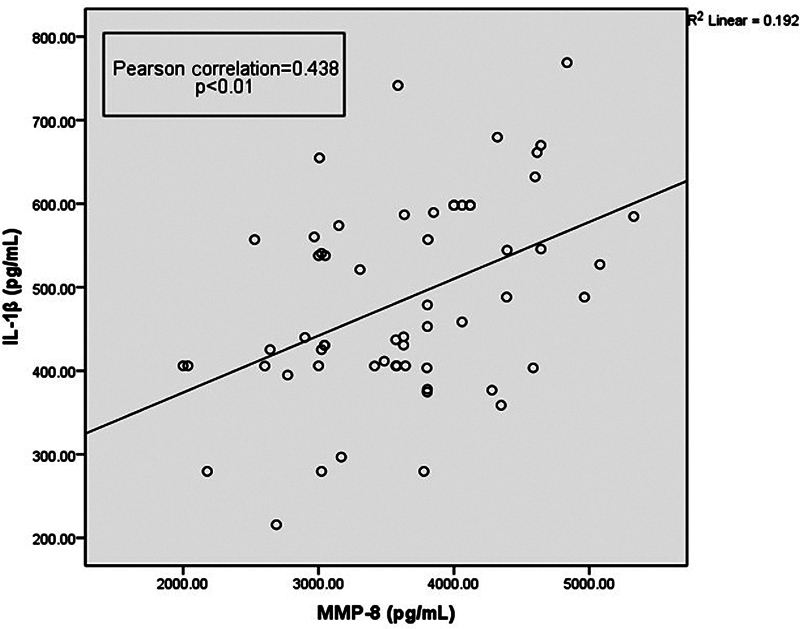
Matrix metalloproteinase-8 (MMP-8) positively correlated with IL-1β levels ( *p* -value < 0.01).

Similarly, the correlation analysis between MMP-8 and IL-1β showed that there is a positive correlation among the two markers in disease groups ( Fig. 9 ). In the previous results, it has been shown that the values of both markers were higher in the two disorders as compared with the healthy controls.

## Discussion

The field of salivary diagnostics is one that is quickly developing and offers clinicians and patients the possibility of quick, noninvasive diagnoses with high accuracy. However, substantial constituent profiling and detailed validation of diagnostic indicators must be done to fully realize the potential of saliva. Due to the asymptomatic trend, periodontal disease is still underdiagnosed at subclinical levels. A strong biomarker is required to assess the status of periodontal disorders at the natal stage. Saliva and GCF have both been used in the past for biomarker assays, but no research has been done on the aforementioned biomarkers in otherwise healthy Pakistani adults.

According to this study, salivary IL-1β and MMP-8 are potential biomarkers to identify periodontitis and gingivitis. Previous reports had shown that these markers, either employed alone or in combination, can distinguish healthy gums from gingivitis and periodontitis. Our study also supports and suggests that both of these markers could identify the severity of periodontal.

We studied the presence of IL-1B for the diagnosis of periodontitis and gingivitis that coincided with the study conducted by Sohail et al in which early diagnosis of oral cancer had been identified by the detection of IL-8 in *naswar* users in a similar population. 23 The use of saliva to evaluate both oral and systemic health had also been described; however, patients with systemic disorders were also included in the previous studies. 24 Our study found that patients with gingivitis and periodontitis exhibited deeper probing depths than healthy controls. 25 26

We found that the average age of periodontitis patients was substantially greater than that of healthy people. It has been reported that age does not have a role in periodontitis. 27 The study found that while some reduction in periodontal attachment and bone is to be expected as people age, age alone does not result in a major loss of periodontal support in a healthy adult. Although significant alveolar bone loss and periodontal attachment are frequent in the elderly, severe periodontitis is rare.

Our study showed that the IL-1B levels were found to be higher in the periodontitis group than in the healthy control and gingivitis groups. Study conducted on salivary IL-1 levels in periodontitis patients and healthy persons noticed the similar tendency. Although there was no statistically significant difference between periodontitis groups, they were significantly higher than those observed in healthy controls. The levels of IL-B in gingivitis were found to be greater than in healthy controls in this investigation. However, when comparing periodontitis groups to healthy controls and gingivitis groups, IL-1B levels were not significantly different. This information matched the clinical measurements as well. The findings of the study reaffirmed the importance of saliva as a sampling method for immunological reasons in periodontal disease. It is suggested that greater IL-1β concentrations could be one of the host–response components connected to clinical symptoms of periodontal disease. 28

MMP-8 levels were found to be greater in gingivitis and periodontitis groups compared with healthy controls. Gingivitis was found to be statistically associated with an elevation in salivary active MMP-8 in a research of adolescents conducted by Romero-Castro et al. 29 This information can give rise to unique and practical kits in practical use for the detection of active form of MMP-8 in oral fluids in periodontitis. 30 31

Our study has shown a positive correlation between MMP-8 and IL-1β in disease groups. Combining salivary biomarkers such as MMP-8 and IL-1B can prove to be a valuable key to diagnose gingivitis and periodontitis. Salivary biomarkers may provide useful extra diagnostic information and could be utilized as tests for periodontal disease screening, prognosis, and prediction, according to recent systematic reviews with high evidence value. In the future, improvements in personalized medicine and proteomic analysis will open the path for novel diagnostic techniques. Its use in dentistry, on the other hand, will be defined by how practitioners integrate technology into their daily clinical practice. 32 33

Particularly in cases of oral diseases including periodontal disease, dental caries, and oral cancer, many saliva indicators are highly sensitive and specific. However, because sophisticated approaches are needed, it is challenging to identify disease-specific biomarkers in whole saliva.

## Limitations

However, when evaluating the results of our study, it is important to take into account two significant limitations. Primarily the small sample size of the study design precluded extrapolating the results to a larger population. Moreover, there are not many studies that examine how combined salivary biomarkers can be used to identify both gingivitis and periodontitis in the literature as a whole. To address these limitations and corroborate the findings, more longitudinal and clinical-trial investigations with a larger sample size are essential.

## Conclusion

It has been argued throughout this work that the routine clinical methods used to identify gingivitis and periodontitis with periodontal probe result in variability among the examiners. The reason for that is the angulation, design, and invasive nature of the diagnostic procedure.

Targeted salivary biomarkers were investigated, and it was observed that these biomarkers can be used to diagnose periodontal disorders accurately. In the current study, the diagnostic precision of salivary MMP-8 and IL-1β as indicators of gingivitis and periodontitis was evaluated. The baseline salivary MMP-8 and IL-1β levels of the periodontal disease groups were greater than those of the healthy subjects. The diagnosis of periodontal disease may benefit from the use of both combinations and individual salivary biomarkers. However, to validate these biomarkers, further methodically thorough research is necessary. Moreover, the prospective use of saliva-based periodontal disease testing opens up a brand-new, intriguing field of noninvasive chairside diagnostics. In this way, this study could serve as a basis for future research into the diagnostic use of salivary biomarkers and, ultimately, the creation of periodontal disease diagnostic kits.

## Future Recommendations

The noninvasive, easy, and affordable sampling are appealing benefits of using saliva banking for the detection of various diseases. Additionally, the use of saliva introduces point-of-care (POC) technology for salivary diagnostics, which could be beneficial in the early diagnosis of systemic and oral diseases like cancer and genetic disorders. 34 “Medical device used to conduct testing outside the laboratory at or near the site of patient care, including the patient's bedside, the doctor's office, and the patient's home,” is how POC technology is categorized. 35 These technologies allow analytes to be identified more rapidly and accurately. Hence, our study refers to the future of periodontology that would be “predictive, preventative, personalized, and participatory periodontology,” often known as the “5Ps age.” 36 The next phase in the evolution of microfluidics, digital microfluidics, looks to have promise for future use in diagnosing periodontal problems and prognosticating periodontal treatment.
